# Understanding the incidence and timing of rabies cases in domestic animals and wildlife in south-east Tanzania in the presence of widespread domestic dog vaccination campaigns

**DOI:** 10.1186/s13567-022-01121-1

**Published:** 2022-12-12

**Authors:** Sarah Hayes, Kennedy Lushasi, Maganga Sambo, Joel Changalucha, Elaine A. Ferguson, Lwitiko Sikana, Katie Hampson, Pierre Nouvellet, Christl A. Donnelly

**Affiliations:** 1 Department of Infectious Disease Epidemiology, Faculty of Medicine, School of Public Health, Imperial College London, London, UK; 2 Department of Statistics, University of Oxford, Oxford, UK; 3 Ifakara Health Institute, Ifakara, Tanzania; 4 Institute of Biodiversity, Animal Health and Comparative Medicine, University of Glasgow, Glasgow, UK; 5 Nelson Mandela African Institution of Science and Technology, Arusha, Tanzania; 6 School of Life Sciences, University of Sussex, Sussex, UK

**Keywords:** Zero by thirty, multi-host, seasonality, Lyssavirus, jackal

## Abstract

The “Zero by 30” strategic plan aims to eliminate human deaths from dog-mediated rabies by 2030 and domestic dog vaccination is a vital component of this strategic plan. In areas where domestic dog vaccination has been implemented, it is important to assess the impact of this intervention. Additionally, understanding temporal and seasonal trends in the incidence of animal rabies cases may assist in optimizing such interventions. Data on the incidence of probable rabies cases in domestic and wild animals were collected between January 2011 and December 2018 in thirteen districts of south-east Tanzania where jackals comprise over 40% of reported rabies cases. Vaccination coverage was estimated over this period, as five domestic dog vaccination campaigns took place in all thirteen districts between 2011 and 2016. Negative binomial generalized linear models were used to explore the impact of domestic dog vaccination on the annual incidence of animal rabies cases, whilst generalized additive models were used to investigate the presence of temporal and/or seasonal trends. Increases in domestic dog vaccination coverage were significantly associated with a decreased incidence of rabies cases in both domestic dogs and jackals. A 35% increase in vaccination coverage was associated with a reduction in the incidence of probable dog rabies cases of between 78.0 and 85.5% (95% confidence intervals ranged from 61.2 to 92.2%) and a reduction in the incidence of probable jackal rabies cases of between 75.3 and 91.2% (95% confidence intervals ranged from 53.0 to 96.1%). A statistically significant common seasonality was identified in the monthly incidence of probable rabies cases in both domestic dogs and jackals with the highest incidence from February to August and lowest incidence from September to January. These results align with evidence supporting the use of domestic dog vaccination as part of control strategies aimed at reducing animal rabies cases in both domestic dogs and jackals in this region. The presence of a common seasonal trend requires further investigation but may have implications for the timing of future vaccination campaigns.

**Supplementary Information:**

The online version contains supplementary material available at 10.1186/s13567-022-01121-1.

## Introduction

‘Zero by 30’ is a global strategic plan to achieve zero human deaths from dog-mediated rabies by 2030 [1]. Despite the existence of safe and effective vaccines, rabies continues to kill an estimated 59,000 people annually [2], primarily in low- and middle-income countries. Transmission from domestic dogs is responsible for 99% of these deaths [3] and domestic dog vaccination is an important component of the Zero by 30 plan. Whilst international standards and guidelines reflecting best practice in rabies control are available, it is recognised that countries need to be supported to adapt these guidelines to their local context to maximise their impact [1]. Ongoing surveillance is vital for measuring the effectiveness of interventions and assessing progress towards the Zero by 30 target [1].

Measuring the impact of interventions is one of the Zero by 30 key objectives. Domestic dog vaccination is considered to directly affect rabies transmission by reducing the size of the susceptible dog population and thus reducing the probability of onward transmission of rabies from a rabid animal. However, assessing and quantifying the real-world impact of domestic dog vaccination on the incidence of animal rabies cases can be challenging. Randomised controlled trials (RCTs) are considered the gold standard for assessing the effects of a treatment or an intervention. However, it is not always ethical or feasible to undertake an RCT and in such instances it may be necessary to use observational studies. Whilst the potential for unmeasured confounding always exists in observational studies, regression methods applied to observational data can be used to draw causal inferences under key assumptions and thus can provide evidence for the impact of interventions. The instantaneous reproduction number (R_t_) may also be used to assess interventions. R_t_ is the average number of secondary cases caused by an infected individual if conditions remain as they are at the time of estimation (*t*). To reduce the incidence of disease, R_t_ needs to be reduced to below one. In the context of rabies, R_t_ has been used to investigate the role of importation of rabid dogs on rabies incidence in the Central African Republic [4].

Another important feature for assessing the effectiveness of control strategies is understanding the local reservoir dynamics. Rabies virus (the causative agent of rabies) is a multi-host pathogen capable of infecting any mammal. Across most of sub-Saharan Africa, including the wildlife-rich Serengeti ecosystem in northern Tanzania, domestic dogs are considered the only population essential for the persistence of rabies [5]. However, in parts of southern Africa, there is evidence of independently maintained cycles of transmission in jackal species (*Canis mesomelas* and *Canis adustus*) and bat-eared foxes (*Otocyon megalotis*) [6–11]. The presence of wildlife species capable either of maintaining rabies independently or contributing to the reservoir of infection could affect the level of domestic dog vaccination required to achieve elimination and/or the likelihood of reintroduction of rabies into domestic dogs following cessation of vaccination campaigns [12, 13]. If wildlife species such as jackals were maintaining rabies independently of domestic dogs, we would expect there to be little association between levels of domestic dog vaccination and the incidence of jackal rabies. Consideration of potential wildlife hosts and their role in rabies transmission is therefore important when planning and assessing interventions.

Many infectious diseases of both humans and animals exhibit seasonality in the incidence of infection [14, 15]. Characterisation of these seasonal patterns can be used to inform interventions. The mechanisms underlying seasonal trends are often complex and not fully understood. Seasonal variability in environmental and climatic conditions, emergence of vectors, host birth rates, contact between hosts due to migration and/or breeding and food availability are all suggested as potential contributors [14]. Seasonality in rabies incidence has been reported in jackals in Namibia and Zimbabwe [16, 17], in red foxes in Europe [18, 19], in skunks and raccoons in North America [20, 21] and in domestic dogs in Peru and Bhutan [22, 23]. If seasonal trends in rabies incidence can be identified at a national or local level, vaccination campaigns could be timed to maximise their impact. A recent modelling study of fox rabies in Europe suggested that autumn vaccination campaigns had a higher probability of achieving elimination than spring vaccinations as they were more likely to reach susceptible juveniles produced by seasonal birth pulses [24].

Canine rabies is endemic across Tanzania, putting people at risk of exposure to this devastating disease [25]. Following a potential rabies exposure (usually from the bite or scratch of an infected animal) it is vital that people immediately receive a course of post-exposure prophylaxis (PEP). Throughout much of sub-Saharan Africa, including Tanzania, access to life-saving PEP is often limited by high cost and supply shortages [26, 27]. Controlling rabies in the animal population(s) responsible for transmitting rabies to people could result in fewer human exposures and reduce demand for PEP. Studies from northern Tanzania report that domestic dogs are responsible for the vast majority of animal rabies cases and human rabies exposures [5, 28].

In this study we use data collected from 13 districts of south-east Tanzania over an eight-year period coincident with the implementation of domestic dog vaccination. Across these 13 districts, jackals comprised an unusually high proportion (> 40%) of the recorded animal rabies cases, prompting questions as to their role in rabies transmission in this part of Tanzania [29]. Three species of jackals (*Canis adustus, Canis mesomelas* and *Canis aureus*) inhabit these districts (personal observation) but there are limited data on their distribution and densities. The aims of this study were to utilise data collected through an observational study to assess the impact of domestic dog vaccination on the incidence of domestic dog and jackal rabies cases and to look for evidence of any temporal or seasonal trends in the incidence of domestic dog and jackal rabies cases.

## Materials and methods

### Study area

**Figure 1:**
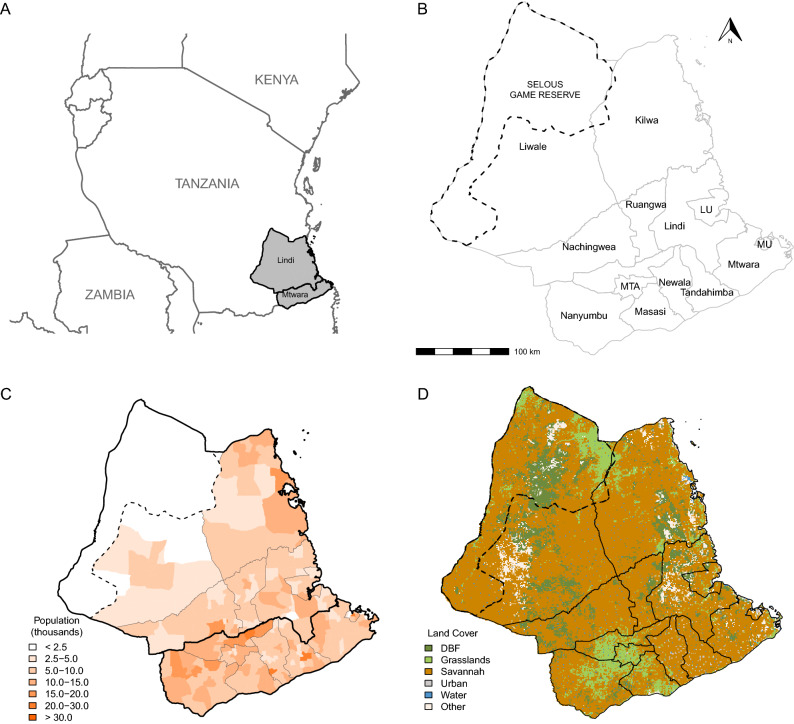
**Study area illustrating selected geographic and human demographic features.** **A** Map of Tanzania showing location of study area (grey) and the regions of Lindi and Mtwara. **B** Districts within the study area. Lindi and Mtwara refer to Lindi Rural and Mtwara Rural respectively. LU, Lindi Urban; MTA, Masasi Township Authority; MU, Mtwara Urban. **C** Population in thousands of people by ward in 2012 (Census year). **D** Predominant land cover types across the study area based on data from 2018. DBF, Deciduous broadleaf forest. The dotted line in **B**–**D** outlines the wildlife protected area of the Selous Game Reserve.

Data on rabies incidence and vaccination coverage were collected from the 13 districts of the Lindi and Mtwara regions of south-east Tanzania between January 2011 and December 2018. The districts are predominantly rural districts with sparse human populations but include three urban districts (Lindi Urban, Mtwara Urban and Masasi Township Authority). There are 1470 villages within the study area with a combined area of 82 668 km^2^ and the human population from the 2012 census is estimated as 2 132 480 [30]. The village is the smallest administrative unit used in this study, followed by ward and then district. A map of the study area is shown in Figure 1.

#### Data

Data are available via the Dryad Digital Repository [31].

#### Human population

Population estimates at either the village, ward or district level were taken from the 2012 census [30] and region-specific growth rates reported in the 2012 census were applied to estimate the populations for each year of the study period.

#### Rabies incidence

Animal-bite victims presenting to health facilities for rabies PEP were recorded. Four health facilities within each district provided PEP and thus data collection was undertaken at these facilities. Contact tracing of all animal-bite victims and owners of biting animals was undertaken to obtain details of each bite incident including the type of biting animal and the global positioning system coordinates of the bite location, as described in [32]. For probable rabies cases in wildlife, details of jackal species were not recorded. Animals were classified as either probable rabies cases or not a case based on information regarding the animal’s behaviour and the circumstances of the bite. The term “probable” is used in accordance with the World Health Organization (WHO) definitions to describe animal rabies cases [3]. That is, a suspected animal rabies case is defined as an animal that presents with any clinical signs of rabies (hypersalivation, paralysis, lethargy, unprovoked abnormal aggression (i.e., biting two or more people or animals and/or inanimate objects), abnormal vocalization and diurnal activity of nocturnal species) and a probable animal rabies case as a suspected case with a reliable history of contact with a suspected, probable or confirmed rabid animal and/or an animal with suspected rabies that is killed, died or disappears within 4–5 days of observation of illness. A confirmed animal rabies case is a suspected or probable animal case confirmed in a laboratory. If additional biting animals or bite victims were identified during investigations, they were also traced, and the owners/bite victims interviewed. The incidence of probable rabid animals, both domestic and wild, was extracted from these data.

#### Mass dog vaccination

Between 2011 and 2016, five mass dog rabies vaccination campaigns were undertaken across all the districts in the study area. The timing of the campaigns varied year-to-year and was determined by the availability of vaccines within a district. The timing of vaccination campaigns was not influenced by the local incidence of rabies. Briefly, the first campaigns were undertaken in 2011 (primarily in February and March), then in 2012 (June), 2014 (primarily June to August), 2015 (August to October) and 2016 (November). Prior to 2011 no rabies vaccination programmes had been undertaken and thus rabies vaccination coverage in the study area was deemed negligible. A central point vaccination strategy was used during the mass dog vaccination campaigns with owners presenting their animals to a temporary vaccination centre established in an accessible village location (as described in [33]). Owners were invited to bring dogs of all ages, including puppies, for vaccination. No domestic dog vaccination campaigns were conducted in 2017 or 2018.

#### Post-vaccination transects

From the third vaccination campaign onwards, post-vaccination transects were conducted in two randomly selected sub-villages in each of a randomly selected subset of villages across the study area. Limits on time and resources prohibited transects being undertaken in all villages and sub-villages. The numbers of vaccinated dogs (marked by temporary collars) and unvaccinated dogs were recorded. Transects were completed by livestock field officers walking or cycling along transect routes on the evening of the campaign day as detailed in [34]. Transect data were recorded at the village level in the rural districts and at ward level in the three urban districts and were available for at least one year of the study for 1146 villages/wards. A total of 2333 entries were recorded over the three years during which the transects were undertaken.

### Dog numbers

Dog numbers were estimated for each of the villages or wards in which post-vaccination transects were performed using the formula:

1 $$\mathrm{Dog \,number }=\frac{Nv}{Pv},\mathrm{ where}, Pv = \frac{Tc}{Tc+Tnc}$$

*Nv* is the number of dogs recorded as being vaccinated during the campaign, *Pv* is the proportion vaccinated according to the transect, *Tc* is number of temporarily collared (vaccinated) dogs observed and *Tnc* the number of uncollared (non-vaccinated) dogs observed in the transect.

Pups are unlikely to be observed during transects but may comprise a substantial proportion of the dog population. A previous study of dog populations in Tanzania by [35] reports an estimated pup: adult ratio of 1:3.81. The dog number estimates calculated using Equation (1) were thus adjusted by this factor to include both adults and pups within the population estimate.

A human: dog ratio (HDR) was calculated and log_10_ transformed for each village/ward and each year for which transect data were available.

Complete data from all three post-vaccination transects were available for 191 villages or wards. Linear regression was undertaken using these data to test for a linear temporal trend in the HDR. No statistically significant trend was identified.

As there was no evidence of a temporal trend in the HDR, data for all years were used to estimate a single HDR for each village or ward within the study area. For the 193/2333 (8.3%) entries where the recorded number of transect-observed collared dogs was greater than the number of vaccinated dogs recorded, the number of collared dogs was set to equal the number of vaccinated dogs. The 371/2333 entries (15.9%) which did not have any dogs recorded as being vaccinated and/or no dogs were recorded in the transect were removed from the analysis. Where the number of collared dogs observed in the transect was recorded as zero, but a non-zero value was recorded for the number of vaccinated dogs [215/2333 entries (9.2%)], the number of collared dogs was set to 0.5. This is a continuity correction analogous to that proposed by Yates for the chi-squared test [36]. Dog numbers were estimated using Equation (1) and adjusted to account for unobserved pups. An HDR was estimated using the census-derived human population estimates and log_10_ transformed.

Bootstrapping was used to estimate the variance of the log_10_ HDR. For each village/ward, for each year, the binomial distribution was used to introduce variation in the number of collared animals observed and the Poisson distribution used to introduce variation into the number of vaccinated dogs recorded. One thousand bootstrap data sets were produced for each village/ward, for each year and used to estimate the variance for each log_10_ HDR. A single weighted mean HDR and associated confidence interval was estimated for each village/ward with the reciprocal of the bootstrap-estimated variance used as the weight.

For any village or ward where missing information precluded estimation of the HDR, the estimated log_10_ HDRs from villages/wards with boundaries adjoining that village or ward were used to estimate a weighted mean for that village/ward.

Following this step, 15 villages still did not have an estimate of the HDR. A ward-level weighted mean was estimated for the wards within which these villages were found and applied to these 15 villages.

Using the estimated HDRs for each village or ward, estimates of dog numbers for each year of the study were produced using the census-derived annual human population estimates. The village and ward estimates of the number of dogs within each district were summed to produce a district-level estimate of dog numbers.

Dog densities were estimated by dividing the estimated number of domestic dogs within a district by the area of the district excluding the wildlife protected area as humans and domestic dogs are not permitted within these areas.

#### Domestic dog vaccination coverage

District-level estimates of dog vaccination coverage for each year that a vaccination campaign took place were estimated using the number of dogs recorded as vaccinated in each vaccination campaign within each district and the district-level estimates of dog numbers. The estimates were assigned to the year that the vaccination campaign took place, irrespective of the timing of the vaccination campaign within that year. The confidence intervals for vaccination coverage were estimated using the reported number of vaccinations undertaken for each district and year and the confidence interval of dog numbers.

#### Land cover

The percentage of each district covered by savannah was calculated using land coverage estimates obtained from Modis [37]. Land coverage was included within the analyses as a potential proxy for habitat suitability for jackal populations. Associations between land use and jackal rabies in Zimbabwe and raccoon rabies in the USA have been previously reported [11, 38]. The presence or absence of a wildlife protected area within a district was also recorded.

### Statistical analysis

The majority of the statistical analyses focus on the incidence of probable rabies cases in domestic dogs and jackals as these account for 495 of the 520 (95.2%) probable animal rabies cases reported over the eight-year period (275 in domestic dogs and 220 in jackals). The remaining 25 probable rabies cases were in domestic cats (10 cases), hyenas (8 cases), honey badgers (5 cases) and leopards (2 cases).

All analyses were undertaken using the R statistical computing language, version 4.1.0. [39].

### Association between domestic dog vaccination coverage and annual rabies incidence

The goal of this analysis was to investigate the impact of domestic dog vaccination coverage and the incidence of rabies cases in domestic dogs and jackals. Negative binomial generalized linear models (GLMs) were used with the outcome variable specified as the observed annual incidence of probable rabies cases (the absolute number per year) recorded in either dogs or jackals within that district. Separate analyses were run for the two species.

Two vaccination coverage estimates were considered—the mean vaccination coverage over the two years prior to cases occurring and the mean vaccination coverage over the three years prior to cases occurring. The vaccination coverage from the year(s) before cases occurred was used in preference to the vaccination coverage in the same year as the cases to ensure that vaccination preceded the reported cases and thus we could rule out reverse causation, namely rabies outbreaks affecting subsequent vaccine uptake in affected areas. The mean vaccination coverage over the previous two or three years was chosen to reflect the population coverage more accurately than single year estimates as demographic processes (births of susceptible animals and deaths of both vaccinated and unvaccinated animals) contribute to temporal changes in population immunity. A maximum of three years prior to the cases occurring was included in the mean percentage vaccination coverage to reflect the expected average lifespan of domestic dogs reported for areas similar to the study regions [40, 41] and the expected duration of immunity from vaccination [42, 43]. A vaccination coverage of zero was assumed for the years prior to 2011 as no mass dog vaccination campaigns had occurred prior to the study and vaccination coverage was deemed negligible.

Variables that were potential confounders of the association between domestic dog vaccination coverage and the incidence of rabies cases or that could be associated with the incidence of rabies cases were included within the models. Confounders need to be included to allow causal inference, whilst factors associated solely with rabies incidence were included to increase the precision of the regression. The variables considered were: area of the district; percentage of land cover in each district classified as savannah; human population size and density; dog population size and density; whether or not the district contained a wildlife protected area and whether the district was classified as either urban or rural. Reliable estimates for jackal populations and densities were not available and thus were not included.

We hypothesize that the availability of susceptible hosts may affect the incidence of rabies cases and thus variables were included that directly reflect the availability of susceptible animal hosts (dog number and density, presence or absence of a wildlife protected area) or may serve as a proxy for host numbers (i.e., human population size and density which may reflect different habitat suitability for different species). Due to the data collection method used, associations between rabies incidence and human population size/density were considered possible due to differences in the potential for human exposure to rabid animals. The percentage area of the district classified as savannah was included in the models where the incidence of jackal rabies cases was the outcome variable as it was hypothesised that savannah may be a suitable habitat for jackals and thus could be associated with jackal population sizes. The categorical classification of districts as urban or rural was included as it was hypothesized that the classification may reflect varying habitat suitability for wildlife species and/or the predominant occupations of the resident human populations which may affect dog-ownership patterns and/or residents’ risk of exposure to rabies and/or may affect the ease of access to a vaccination centre.

For each of the four scenarios evaluated (two different outcome variables (incidence of rabies cases in dogs or jackals) and two vaccination coverage scenarios (mean from two years or three years before cases occurred), four models were fitted. These are outlined in Table 1. GLMs were fitted within the MASS package in R [44].

**Table 1:** **Structure of the generalized linear models analysed**.

Model number	Variable of interest	Covariates
*Domestic dog models*		
Dog model 1	Vaccination coverage (%)^a^	
Dog model 2	Vaccination coverage (%)^a^	Urban/rural
Dog model 3	Vaccination coverage (%)^a^	Urban/rural + human population + dog population + area + presence of protected area
Dog model 4	Vaccination coverage (%)^a^	Urban/rural + human population density + dog population density + area + presence of protected area
*Jackal models*		
Jackal model 1	Vaccination coverage (%)^a^	
Jackal model 2	Vaccination coverage (%)^a^	Urban/rural
Jackal model 3	Vaccination coverage (%)^a^	Urban/rural + human population + dog population + area + presence of protected area + savannah (%)
Jackal model 4	Vaccination coverage (%)^a^	Urban/rural + human population density + dog population density + area + presence of protected area + savannah (%)

In the domestic dog models, the outcome variable is the annual incidence of probable rabies cases in domestic dogs, whilst in the jackal models the outcome variable is the annual incidence of probable rabies cases in jackals.

^a^Vaccination coverage was included either as the mean coverage over the two years prior to cases occurring or over the three years prior to cases occurring.

#### Seasonality and trend

Generalized additive models (GAMs) with a Poisson error distribution were fitted to the monthly incidence data for probable rabies cases in jackals and dogs to investigate the presence of within-year seasonality and an overall temporal trend for the whole period. A negative binomial error distribution was also considered but model checks suggested the Poisson error distribution was appropriate for these monthly incidence data. A selection of models allowing for common or species-specific smooths for one or both components were evaluated. Gaussian process smooths were used to estimate the overall trend and cyclic cubic regression splines for the seasonality. The upper limits for the dimension of the basis functions were set at 10 for the overall trend and 12 for the seasonality. The best-fitting model was selected using Akaike’s Information Criterion (AIC) and evaluation of the deviance explained. GAMs were fitted using the mgcv package [45].

#### Instantaneous reproduction number

R_t_ was estimated using daily incidence data for probable animal rabies cases and an estimate for the serial interval distribution of rabies in dogs using the EpiEstim package [46]. The serial interval, defined as the interval between the onset of clinical signs in a primary case to the onset of clinical signs in a secondary case, was estimated from a long-term contact tracing study in domestic dogs in the Serengeti district of northern Tanzania as detailed in [29]. The serial interval distribution was best characterised by a lognormal distribution with mu and sigma parameters of 2.80 and 0.97 respectively, corresponding to a mean serial interval of 26.3 days with a standard deviation of 32.7 days. The same distribution was assumed for both domestic dogs and jackals as serial interval data for jackals were not available. The EpiEstim package requires the user to select a time window over which to estimate the R_t_, with longer time windows providing more stable estimates but being less responsive to temporal changes in R_t_. Given the long serial interval of rabies and sparse case incidence during the latter years of the study, a window of 120 days was used with sensitivity analysis undertaken using windows of 60 and 180 days. The mean and standard deviation of the prior distribution of R_t_ were both set at 1.0 to reflect the endemicity of canine rabies in the region. R_t_ was estimated for all species combined (to reflect the overall transmission of rabies since there is evidence of both within- and between-species transmission [29]) and, for comparison, independently for both jackals and domestic dogs (which would reflect a situation where rabies was being maintained independently by each species) using all cases within the study area. As local rabies transmission is more frequent than long-distance transmission [29, 32], a sensitivity analysis using a subset of cases that were in a smaller geographic area and where each case was located within 10 km of another case was also undertaken. This subset consisted of 426 cases located within the southern part of the study area (Additional file 1). In addition, a sensitivity analysis was performed where both the mean and standard deviation of the prior distribution of R_t_ were set at 1.2 to reflect estimates for the basic reproduction number (R_0_) (the average number of new infections a single typically infectious individual creates in a completely susceptible population) for rabies in domestic dogs that are reported within the literature [32, 47].

## Results

Over the eight-year study period, 520 probable animal rabies cases were recorded. Overall district-level estimates of the HDR varied between 22.2 to 107.2 although substantial heterogeneity was apparent in the estimates at the individual village/ward level (Table 2). Estimates of annual district-level vaccination coverage ranged from 0 to 56.8% (Additional file 2). The median of the non-zero coverage estimates was 36.1%. Domestic dog density estimates ranged from 0.1 to 8.9 dogs per km^2^.

**Table 2:** **Human and dog demography for each of the study districts**.

District	Human population 2012 (Census year)	Dog number estimates 2012 (95% confidence intervals)	District-level HDR estimate	Median of individual village/ward HDR estimates (Inter-quartile range)	Number of wards or villages within district used for estimation of HDR
Kilwa (excluding island of Songosongo)	187 718	5122 (4434–5810)	36.6	45.6 (24.3–69.6)	101
Lindi Rural	194 143	4483 (3998–4969)	43.3	51.6 (29.4– 83.9)	134
Lindi Urban	78 841	1934 (1769–2099)	40.8	44.7 (35.9–52.1)	18
Liwale	91 380	2192 (1563–2821)	41.7	62.8 (35.7–104.9)	76
Masasi	247 993	6178 (5480–6876)	40.1	42.0 (26.3–73.9)	147
Masasi Township Authority	102 696	4625 (4133–5117)	22.2	22.0 (15.6–23.9)	12
Mtwara Rural	228 003	4133 (3185–5080)	55.2	84.0 (51.2–137.8)	156
Mtwara Urban	108 299	1412 (1182–1642)	76.7	86.6 (53.3–118.7)	15
Nachingwea	178 464	5036 (4852–5221)	35.4	40.7 (24.8–62.1)	118
Nanyumbu	150 857	2436 (2187–2686)	61.9	65.5 (39.4–109.4)	89
Newala	205 492	4025 (3131–4920)	51.1	88.8 (38.8–141.2)	153
Ruangwa	131 080	2831 (2649–3014)	46.3	51.4 (33.2–66.3)	89
Tandahimba	227 514	2123 (1927–2319)	107.2	137.3 (73.6–246.1)	155

Wards were used for estimation of dog numbers within urban districts (Lindi Urban, Mtwara Urban and Masasi Township Authority) and villages used in all other districts. The district-level human: dog ratio (HDR) shown was calculated by dividing the human population estimate in column two with the point estimate for dog numbers in column three.

### Association between domestic dog vaccination coverage and annual rabies incidence

#### Rabies incidence in domestic dogs

An increase in domestic dog vaccination coverage was strongly and consistently associated with a decrease in the incidence of domestic dog rabies cases in all models when included as either the mean percentage vaccination coverage from the two years before cases occurred or the three years before cases occurred. When included as the mean vaccination coverage from two years before cases occurred, all four models produced estimates of between a 78.0 and 79.3% reduction in domestic dog rabies cases associated with a 35% increase in domestic dog vaccination coverage. (The association with a 35% increase in vaccination coverage is reported to represent the median of the vaccination coverage achieved.) The 95% confidence intervals (CIs) for the estimates from all four models ranged from a 61.2 to 88.3% reduction in cases associated with a 35% increase in vaccination coverage.

When including vaccination coverage as the mean percentage coverage from the three years before cases occurred, all four models produced estimates of between an 84.2 and 85.5% reduction in domestic dog rabies cases associated with a 35% increase in domestic dog vaccination coverage, with the 95% CIs ranging from a 70.8 to 92.2% reduction.

#### Rabies incidence in jackals

An increase in domestic dog vaccination coverage was strongly and consistently associated with a decrease in the incidence of jackal rabies cases in all models when included as either the mean percentage vaccination coverage from the two years before cases occurred or the three years before cases occurred. When included as the mean vaccination coverage from two years before cases occurred, all four models produced estimates of between a 75.3 and 79.3% reduction in jackal rabies cases associated with a 35% increase in domestic dog vaccination coverage, with the 95% CIs ranging from a 53.0 to 88.8% reduction.

When including vaccination coverage as the mean percentage coverage from the three years before cases occurred, all four models produced estimates of between an 89.5% and 91.2% reduction in jackal rabies cases associated with a 35% increase in domestic dog vaccination coverage, with the 95% CIs ranging from a 78.0 to 96.1% reduction.

**Figure 2:**
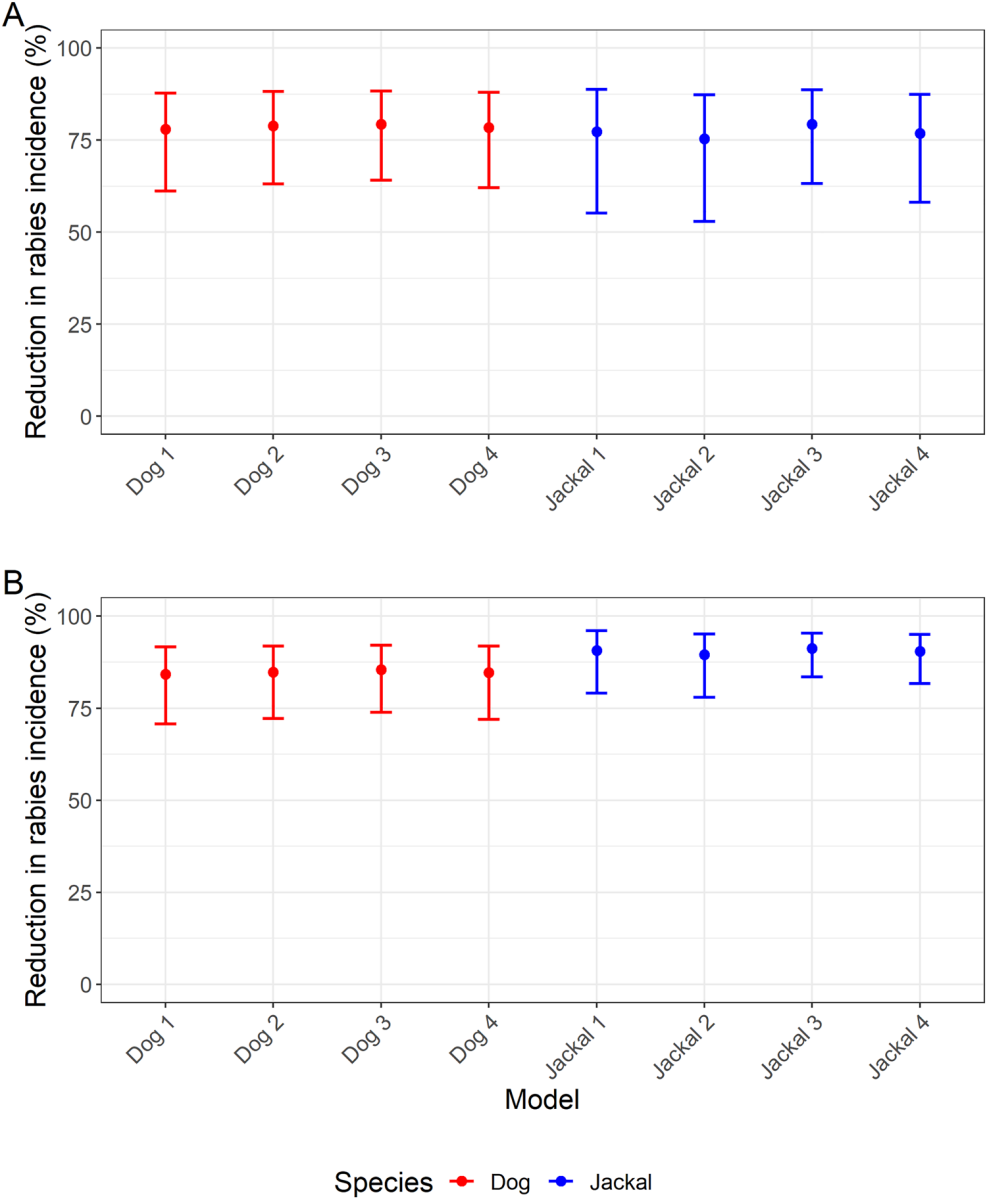
**Estimated effect of domestic dog vaccination coverage on rabies incidence.** Estimated reduction in rabies incidence in domestic dogs and jackals associated with a 35% increase in vaccination coverage. In the domestic dog models, the outcome variable is the annual incidence of probable rabies cases in domestic dogs, whilst in the jackal models the outcome variable is the annual incidence of probable rabies cases in jackals. **A** Results from models containing the mean domestic dog vaccination coverage from two years before cases occurred. **B** Results from models containing the mean domestic dog vaccination coverage from three years before cases occurred. See Table 1 for full specification of each model.

The results of the estimates of the effect of vaccination coverage from the four sets of models are shown in Figure 2, whilst the results of all the models are available in Additional files 3, 4, 5, 6.

Within all the jackal models examined, the presence of a protected area within a district was associated with a statistically significant decrease in the incidence of jackal rabies cases. An increase in the percentage of a district classified as savannah was always associated with an increase in the incidence of jackal rabies cases and whilst the results were not always statistically significant at the 5% level, those that were not approached significance in all cases.

### Seasonality and trend

**Figure 3:**
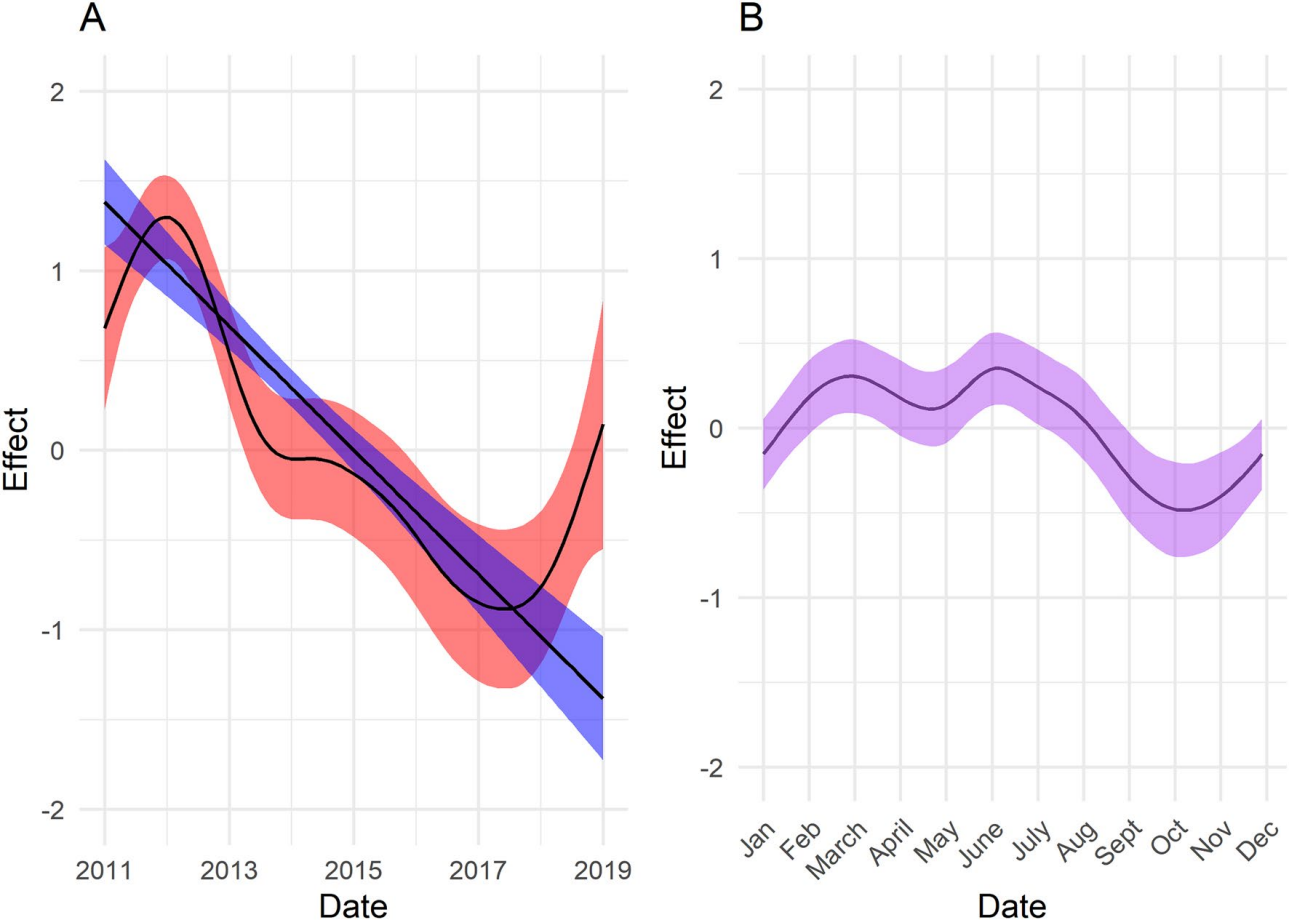
**The overall and seasonal trend components of the best-fitting generalized additive model to rabies incidence.** **A** Relative effect of overall trend in incidence in dogs (red) and jackals (blue) over the course of the study period. **B** Relative effect of common seasonal trend for both species. In both panels the solid line represents the estimate and the shaded area represents the 95% CI of those estimates.

**Figure 4:**
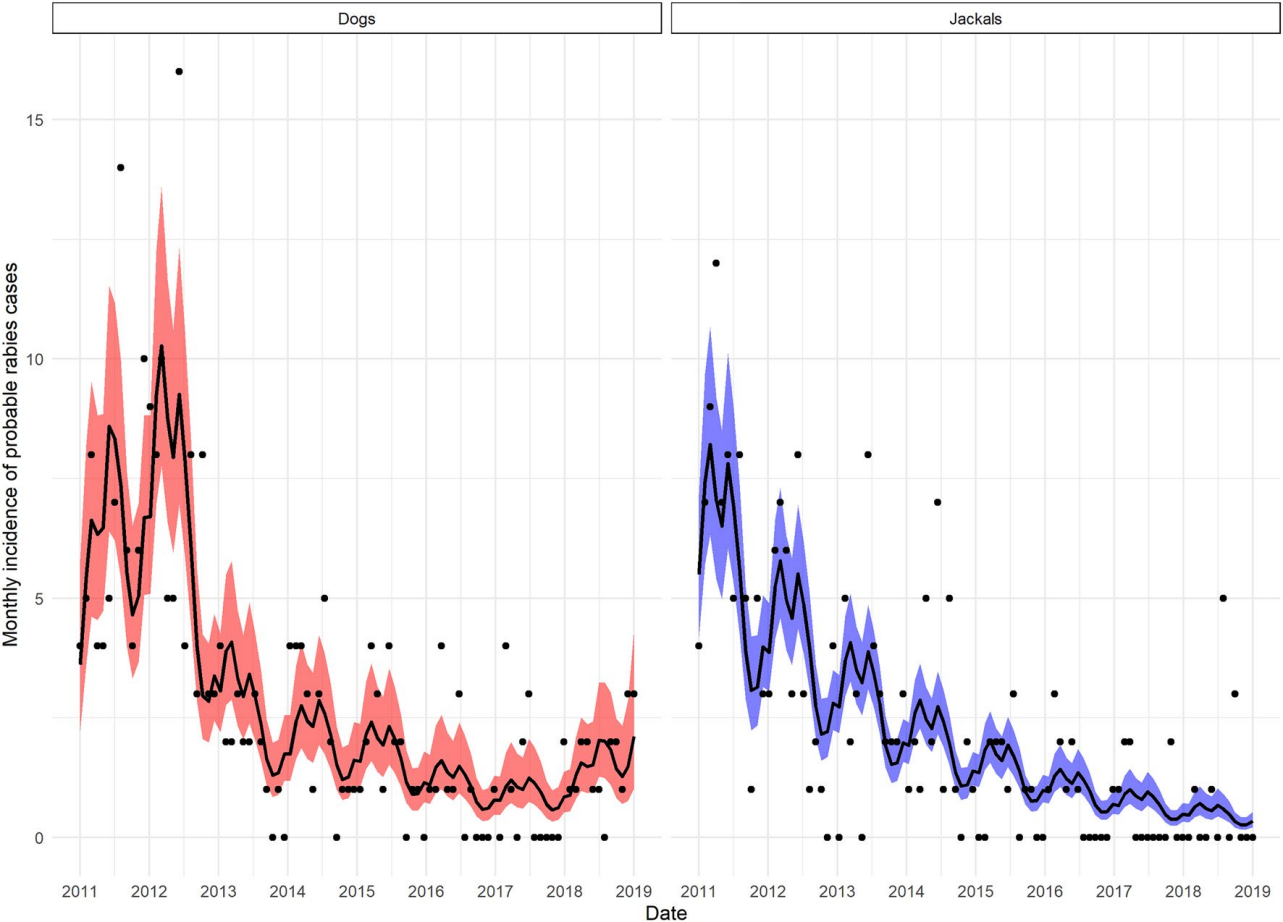
**Monthly incidence data for probable rabies cases in dogs and jackals and associated model fits.** Data are shown as dots. Model fits are from the best-fitting generalized additive model with a single seasonal component but separate components for the overall trend for each species. The solid line represents the model for each species and the shaded area represents the 95% CI of those estimates.

The best-fitting GAM to the monthly incidence data for probable rabies cases in domestic dogs and jackals contained a species-specific overall temporal trend for the study period, but a common seasonality for both species. The adjusted R-squared for the best-fitting model was 0.63 with 60.1% of deviance explained. Model outcomes suggested a decline in monthly incidence in both dogs and jackals over the study period (2011–2018) except for a small increase in incidence in domestic dogs at the end of the study period (2017–2018). The seasonal trend suggested that for both species, monthly incidence was higher from February to August and lower from September to January. The seasonal and overall trend components from the best-fitting model can be seen in Figure 3 whilst the model projections compared to the observed data are shown in Figure 4.

### Instantaneous reproduction number

**Figure 5:**
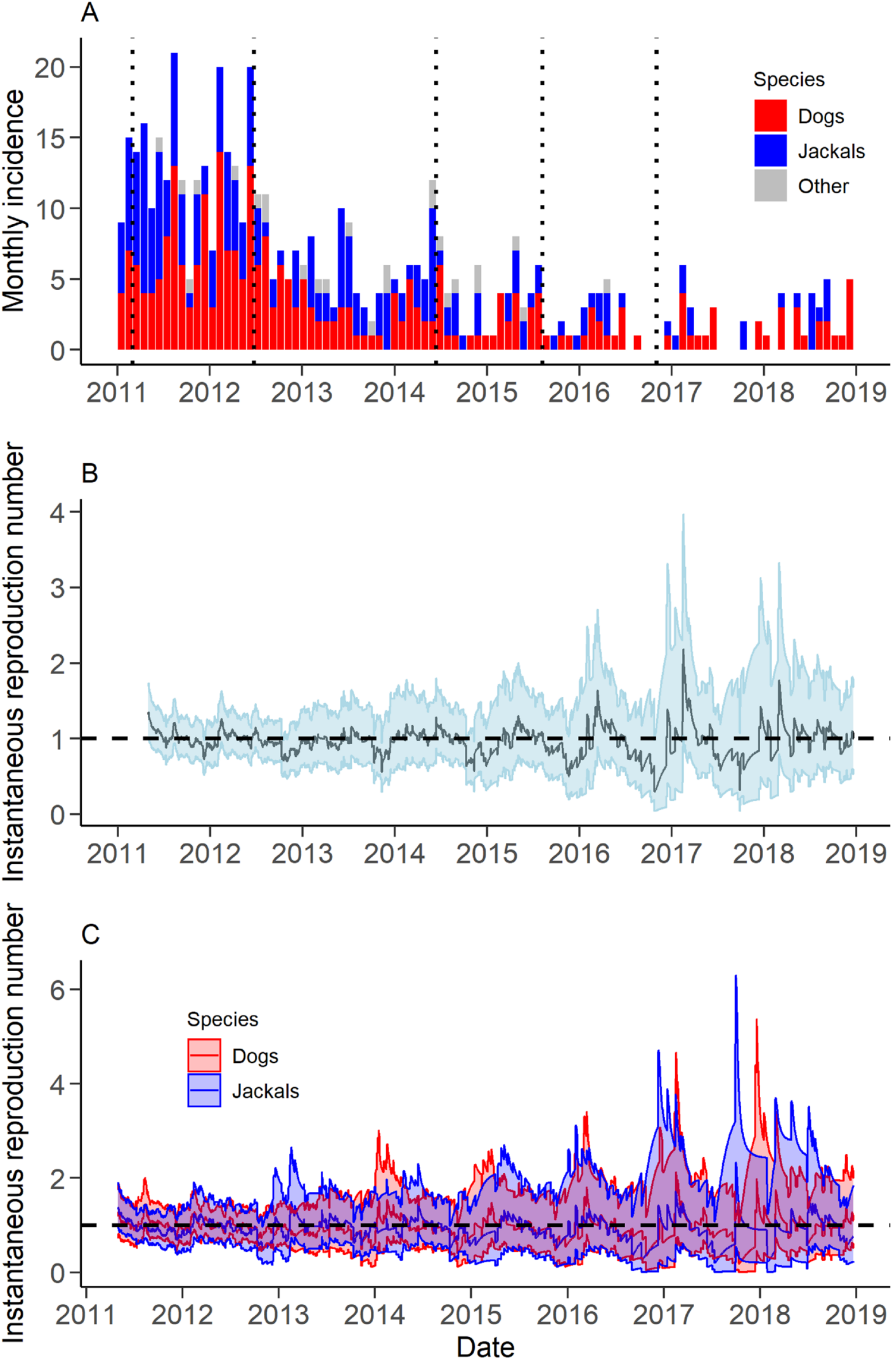
**Monthly incidence of probable rabies cases and associated estimates of the instantaneous reproduction number (R**_**t**_**).** **A** Thirty-day incidence of probable rabies cases in all species. Dotted horizontal lines represent timing of vaccination campaigns. **B** Estimate of R_t_ for all species. The solid line depicts the median estimate with the 95% credible interval represented by the shaded area. **C** Median R_t_ estimates for dogs (red) and jackals (blue). The solid lines depict the median estimates with the 95% credible interval represented by the shaded areas. Horizontal dotted line represents an R_t_ of 1 (**B** and **C**).

Over the course of the study period, the estimated R_t_ oscillated around a value of one for all species combined and for dogs and jackals independently (Figures 5B and C). Estimates of R_t_ were available for 2970 days throughout the study period. For more than 99% of these estimates the 95% credible interval included one for all scenarios (all species combined, dogs only and jackals only). Uncertainty in the R_t_ estimates increased over the latter part of the study when the monthly incidence was lower (Figure 5A). A temporal pattern evident within the R_t_ estimates in all species combined and in the separate estimates for dogs and jackals appeared to follow the same pattern as the seasonality estimated by the best-fitting GAM. Similar results were obtained for the scenarios explored in the sensitivity analyses (Additional file 7).

## Discussion

We find clear evidence for a decrease in incidence of probable rabies cases in both dogs and jackals in south-east Tanzania over an eight-year period, coincident with implementation of widespread domestic dog vaccination campaigns. The results of the regression analyses provide evidence to support the use of domestic dog vaccination in controlling rabies in both domestic dog and jackal populations in this area, thereby reducing the threat to humans and driving progress towards the Zero by 30 target. The finding of a seasonal trend in incidence common to both species warrants further investigation to identify whether future dog vaccination campaigns could be timed to maximise their impact.

Whilst we acknowledge the observational nature of the data, the results presented here are consistent across species and model formulations and add to the existing evidence base to provide a compelling argument for domestic dog vaccination and its inclusion as a key component of the Zero by 30 strategic plan [1, 3, 48–51]. Whilst an association between increasing domestic dog vaccination and decreasing rabies incidence in domestic dogs would be expected due to both direct and indirect protection of dogs, the association is more notable in jackals. The reported associations between domestic dog vaccination and the incidence of rabies in both dogs and jackals suggests that it is unlikely that jackals maintain rabies in a transmission cycle distinct to that of domestic dogs. Instead, our results suggest that rabies may transmit freely between domestic dogs and jackals, but that domestic dogs are the essential population for maintenance of rabies in this region and cause frequent introductions in wildlife populations where species co-exist. If this is the case, reducing the rabies burden in dogs could also have important conservation implications for wildlife as endangered species such as African wild dogs and Ethiopian wolves have been shown to be vulnerable to rabies outbreaks [52, 53].

Whilst the post-vaccination transects allowed area-specific estimates of dog numbers, there were challenges in estimating the population-level of immunity to rabies in the domestic dog population. Population-level immunity is a function of multiple factors, including vaccination coverage, duration of vaccine-induced immunity (which may vary by age and vaccination history [54, 55]) and population turn-over (which is likely to be high within the study area [40]). Whilst waning immunity and population turnover were not incorporated in the models, vaccination coverage was estimated using detailed records from vaccination campaigns and post-vaccination transects. Attempts to overcome some of the challenges associated with point estimates of vaccination coverage were made by estimating mean coverage for up to three years before cases occurred, thereby smoothing out the otherwise marked fluctuations in yearly coverage.

Demonstrating causality from observational data is challenging. Whilst we have included many potential confounders in the analyses presented, it is impossible to rule out unmeasured confounding which could bias our effect estimates. However, the consistency of the effect estimates within each group of models, including the model containing only vaccination coverage, the narrow confidence intervals and large effect sizes add considerable weight to our results and conclusions. Importantly, there is a plausible mechanism between increasing vaccination coverage and reducing disease incidence.

An interesting observation from the results of the jackal models was the association between the presence of a protected area within a district and a reduction in incidence of probable jackal rabies cases and the association between an increase in the percentage of the district that was savannah and the increase in jackal rabies cases. These variables were included within the models as a possible proxy for the number of jackals. Domestic dogs are excluded from protected areas and thus the presence of a protected area may also affect the levels of contact between domestic dogs and jackals. However, these variables were not the main variables of interest in the models and thus we must be cautious in over-interpreting these associations. Further investigation into the roles of protected areas and the size and distribution of jackal populations is warranted to improve our understanding of the transmission dynamics in jackals and domestic dogs in this area.

The common seasonal trend identified in both species may be relevant for the timing of future vaccination campaigns and warrants further investigation. Vaccination campaigns typically took one or two months to complete and whilst not undertaken at the same time every year, in 2012, 2014 and 2015 most took place between June and September (Additional file 2), which coincides with the period of the greatest seasonal declines (Figure 2). The pulsed nature of campaigns may thus have contributed to the observed seasonality. Future analysis of the temporal trends in incidence following cessation of vaccination campaigns (from November 2016) may shed further light on the influence of the pulsed vaccination campaigns on seasonality.

Other reasons suggested for seasonal trends in infectious disease incidence include climatic conditions, birth pulses, seasonal fluxes in contact due to migration and/or breeding and food availability [14]. Whilst domestic dogs do not typically exhibit seasonal reproduction, under certain environmental conditions whelping may be concentrated at particular times of year [56]. A study from northern Tanzania reported a peak in domestic dog litters in July and August [40]. Seasonality in jackal reproduction has also been reported and whilst the exact timing is likely to vary by species and latitude, *C. mesomelas* have been reported to whelp in July–September in Tanzania [56] whilst in Zimbabwe whelping of both *C. mesomelas* and *C. adustus* is reported in September–October [57]. Assuming an eight- to ten-week period before pups emerge [56]) would result in an increase in the susceptible dog population in September–December and an increase in the susceptible jackal population in November–January. The observed seasonal increase in incidence between October and March could plausibly be in part due to increases in susceptible animals during this period. Additional information regarding the species of biting jackals and the reproductive and movement ecology of dogs and jackals in this area may enhance our understanding of the observed seasonality in rabies incidence. Other potential confounding factors may however also exist. For example, as the data collection process uses animal bites to identify probable rabies cases, factors that increase animal-human contact, such as farming practices linked to seasonal weather patterns could result in an increase in opportunities for bites to occur.

The extended time series of data and consistent reporting effort enabled the identification of clear temporal trends in rabies incidence. Contact tracing allowed us to identify a much greater proportion of cases than would have been possible using official case reports alone and identification of individuals (and thus identification of the biting animal) that did not present to health facilities, but our study still lacked laboratory-confirmed rabies cases. This was predominantly due to delays in reporting meaning that on follow-up the animal was lost or the carcass decomposed and it was not possible to collect samples. However, a clinical diagnosis of rabies has been reported to be a reliable method in the absence of laboratory capacity [5]. Whilst animals infected with canine distemper virus may also present with neurological signs [58, 59], evidence of increased aggression and tendency to bite, which is used to identify cases in this study, is more typically reported as a clinical sign of rabies [60, 61].

The estimates of R_t_ are difficult to interpret. The R_t_ estimates of one, or just below one, could be compatible with effective interventions or simply reflect endemic disease. However, the consistency and strength of the estimates in the regression analysis provides convincing evidence for the effectiveness of domestic dog vaccination and the low incidence of probable rabies cases, especially in latter years, limits the accuracy of the R_t_ estimates. Future analysis to estimate R_t_ in the absence of ongoing vaccination may provide further insight.

This study supports the use of domestic dog vaccination to reduce animal rabies cases and provides further support for its use as an integral component of the Zero by 30 strategy. The results suggest that jackals are unlikely to maintain rabies independent of domestic dogs and that domestic dog vaccination may benefit wildlife populations as well as importantly reducing human exposures and deaths due to wildlife rabies in this area of Tanzania. Finally, a common seasonal trend in the incidence of rabies in both dogs and jackals suggests that it may be possible to optimise the timing of vaccination strategies in this region to achieve the maximum impact.

## Supplementary Information

**Additional file 1. Location of the probable animal rabies cases used within the subset analysis.** Probable animal rabies cases are shown as black diamonds and include all species. The cases considered within the subset analysis are those located in the region outlined in blue. All cases included within the subset are within 10 km of another case.

**Additional file 2. Vaccination coverage estimates.** Estimates for each district for each year during which vaccination campaigns were undertaken. Details of the timings of the vaccination campaign are given for each year.

**Additional file 3. Results of generalized linear models for the incidence of probable domestic dog rabies cases from models containing the mean domestic dog vaccination coverage from two years before cases occurred.** The 95% confidence intervals for the estimates are shown in square brackets [].

**Additional file 4. Results of generalized linear models for the incidence of probable domestic dog rabies cases from models containing the mean domestic dog vaccination coverage from three years before cases occurred.** The 95% confidence intervals for the estimates are shown in square brackets [].

**Additional file 5. Results of generalized linear models for the incidence of probable jackal rabies cases from models containing the mean domestic dog vaccination coverage from two years before cases occurred.** The 95% confidence intervals for the estimates are shown in square brackets [].

**Additional file 6. Results of generalized linear models for the incidence of probable jackal rabies cases from models containing the mean domestic dog vaccination coverage from three years before cases occurred.** The 95% confidence intervals for the estimates are shown in square brackets [].

**Additional file 7. Estimates of the instantaneous reproduction number (R**_**t**_**) for all species combined under different scenarios.** The solid line depicts the median estimate with the 95% credible interval estimates represented by the shaded area. The time window used for estimation of R_t_ are **A** 60 days, **B** 180 days, **C** and **D** 120 days. The values for the mean and standard deviation of the prior distribution are both set at 1.0 in **A**, **B** and **D** and at 1.2 in **C**. All data are used for estimation in **A**–**C** (*n* = 520 cases) whilst a subset of data where all cases are less than 10 km from another case were used in **D** (*n* = 426 cases).

## Data Availability

The datasets supporting the conclusions of this article are available in the dryad repository https://doi.org/10.5061/dryad.63xsj3v5m. The code used for the analyses are available in the public GitHub repository https://github.com/sarahhayes/rabies_vacc_seasonality.
