# Supplementary figures and images for: Understanding the incidence and timing of rabies cases in domestic animals and wildlife in south-east Tanzania in the presence of widespread domestic dog vaccination campaigns
Source: Vet Res. 2022 Dec 12;53:106. doi: 10.1186/s13567-022-01121-1 (PMC9743725; doi:10.1186/s13567-022-01121-1)

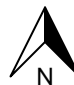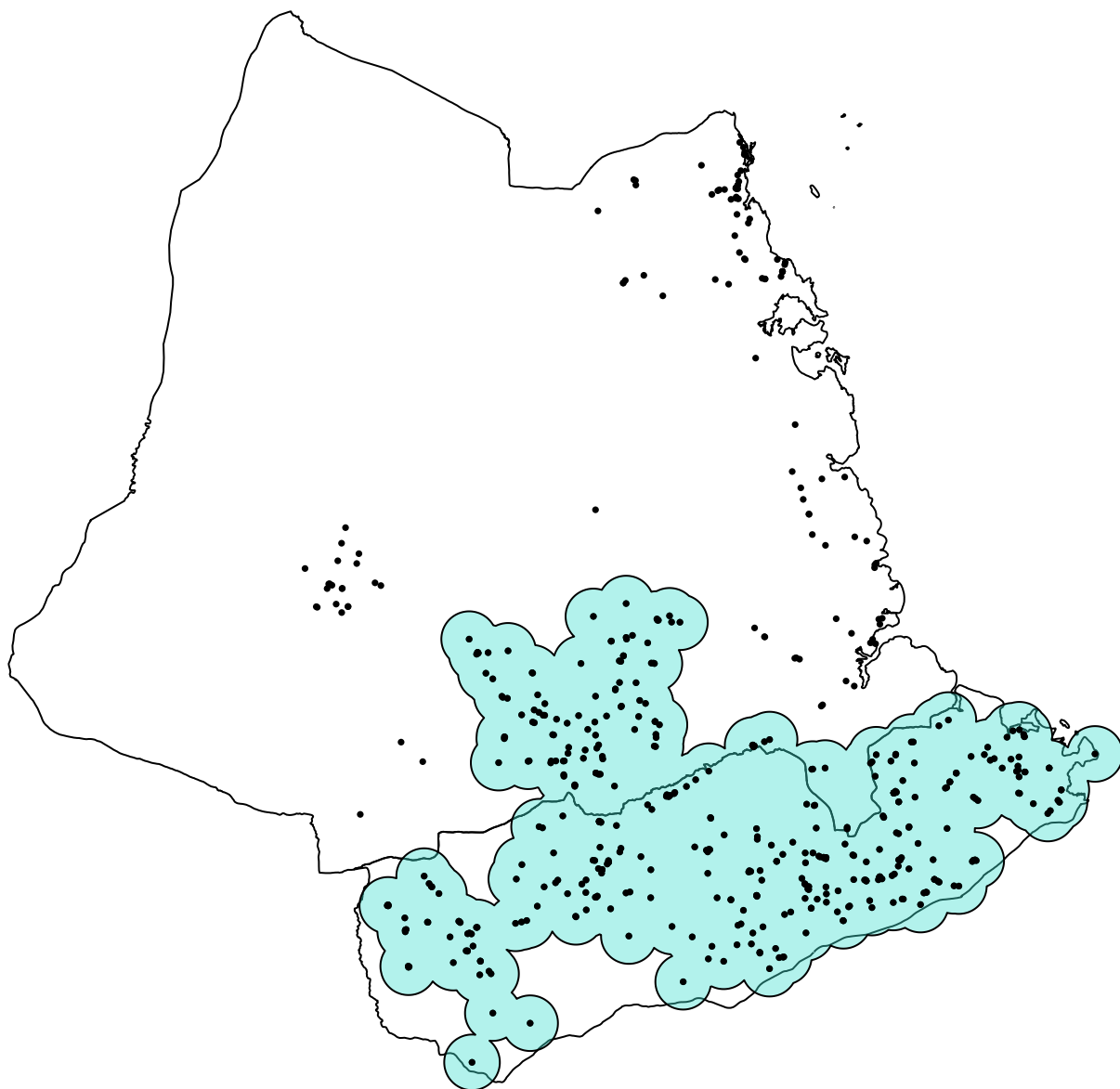

100 km

Supplement: Supplementary file 1 — Additional file 1. Location of the probable animal rabies cases used within the subset analysis. Probable animal rabies cases are shown as black diamonds and include all species. The cases considered within the subset analysis are those located in the region outlined in blue. All cases included within the subset are within 10 km of another case. [file 13567_2022_1121_MOESM1_ESM.pdf]

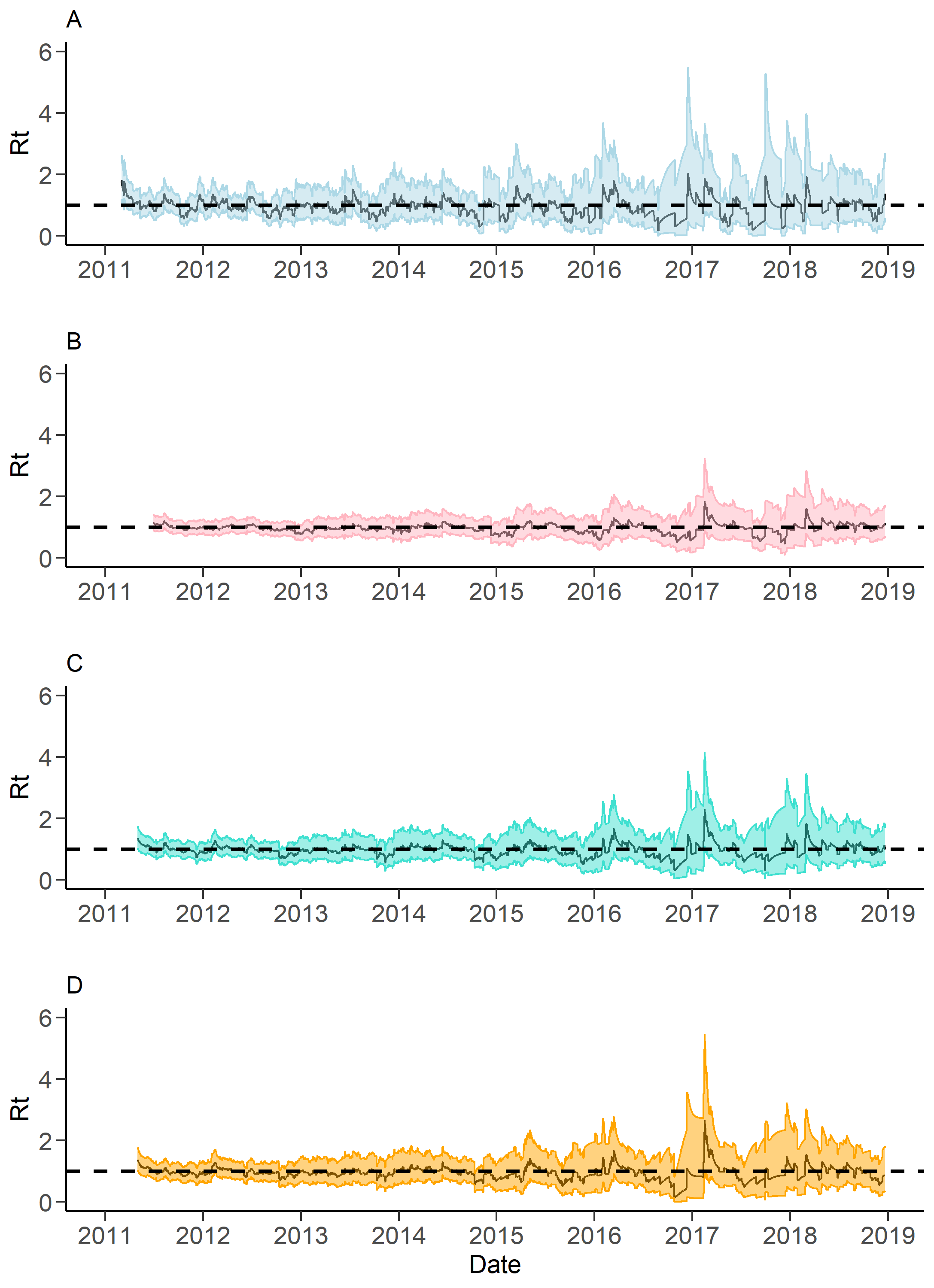

Supplement: Supplementary file 7 — Additional file 7. Estimates of the instantaneous reproduction number (Rt) for all species combined under different scenarios. The solid line depicts the median estimate with the 95% credible interval estimates represented by the shaded area. The time window used for estimation of Rt are A 60 days, B 180 days, C and D 120 days. The values for the mean and standard deviation of the prior distribution are both set at 1.0 in A, B and D and at 1.2 in C. All data are used for estimation in A–C (n = 520 cases) whilst a subset of data where all cases are less than 10 km from another case were used in D (n = 426 cases). [file 13567_2022_1121_MOESM7_ESM.png]
