# Supplementary material for: Understanding the incidence and timing of rabies cases in domestic animals and wildlife in south-east Tanzania in the presence of widespread domestic dog vaccination campaigns
Source: Vet Res. 2022 Dec 12;53:106. doi: 10.1186/s13567-022-01121-1 (PMC9743725; doi:10.1186/s13567-022-01121-1)
Supplement: Supplementary file 2 — Additional file 2. Vaccination coverage estimates. Estimates for each district for each year during which vaccination campaigns were undertaken. Details of the timings of the vaccination campaign are given for each year. [file 13567_2022_1121_MOESM2_ESM.docx]

**Vaccination coverage estimates.** Estimates for each district for each year during which vaccination campaigns were undertaken. Details of the timings of the vaccination campaign are giving for each year.

| District | Percentage Vaccination Coverage  [95% Confidence interval] | | | | |
| --- | --- | --- | --- | --- | --- |
|  | **2011**  (Primarily February and March with small amount in June) | **2012**  (June) | **2014**  (Primarily June – August with small amount in March, September and November) | **2015**  (August – October) | **2016**  (November) |
| Kilwa | 25.1  [22.1 – 28.9] | 38.3  [33.7 – 44.2] | 40.7  [35.8 – 47.0] | 39.0  [34.4 – 45.1] | 49.9  [44.0 – 57.7] |
| Lindi Rural | 29.4  [26.5 – 33.0] | 38.9  [35.1 – 43.7] | 25.2  [22.7 – 28.2] | 37.3  [33.7 – 41.9] | 35.3  [31.8 – 39.6] |
| Lindi Urban | 56.1  [51.7 – 61.3] | 50.0  [46.1 – 54.7] | 47.2  [43.5 – 51.6] | 56.8  [52.3 – 62.1] | 53.4  [49.2 – 58.4] |
| Liwale | 25.3  [19.7 – 35.5] | 35.6  [27.6 – 49.9] | 28.5  [22.2 – 40.3] | 26.3  [20.5 – 36.9] | 19.0  [14.7 – 26.6] |
| Masasi | 37.2  [33.4 – 41.9] | 35.4  [31.8 – 40.0] | 37.1  [33.4 – 41.9] | 36.1  [32.4 – 40.7] | 29.9  [26.9 – 33.7] |
| Masasi Township Authority | 5.8  [5.2 – 6.5] | 27.2  [24.6 – 30.5] | 46.6  [42.2 – 52.2] | 46.3  [41.9 – 51.8] | 18.0  [16.3 – 20.2] |
| Mtwara Rural | 11.9  [9.7 – 15.4] | 21.0  [17.0 – 27.2] | 20.3  [16.5 – 26.4] | 25.2  [20.5 – 32.7] | 20.0  [16.3 – 26.0] |
| Mtwara Urban | 39.8  [34.3 – 47.6] | 27.1  [23.3 – 32.4] | 37.3  [32.1 – 44.6] | 35.9  [30.8 – 42.9] | 38.1  [32.8 – 45.6] |
| Nachingwea | 39.7  [38.3 – 41.2] | 54.3  [52.4 – 56.4] | 55.1  [53.1 – 57.2] | 50.6  [48.8 – 52.5] | 45.2  [43.6 – 46.9] |
| Nanyumbu | 26.1  [23.7 – 29.1] | 30.6  [27.8 – 34.1] | 51.3  [46.6 – 57.2] | 44.1  [40.0 – 49.1] | 45.5  [41.3 – 50.7] |
| Newala | 25.2  [20.7 – 32.5] | 27.6  [22.7– 35.5] | 35.5  [29.1 – 45.7] | 18.7  [15.3 -24.0] | 16.6  [13.6 – 21.4] |
| Ruangwa | 40.9  [38.4 – 43.7] | 40.1  [37.6 – 42.8] | 37.8  [35.5 – 40.4] | 48.4  [45.5 – 51.8] | 56.0  [52.6 – 59.8] |
| Tandahimba | 28.4  [26.0 – 31.2] | 29.1  [26.7 – 32.1] | 35.0  [32.1 – 38.6] | 37.6  [34.4 – 41.4] | 36.0  [32.9 – 39.6] |
