# Supplementary material for: Understanding the incidence and timing of rabies cases in domestic animals and wildlife in south-east Tanzania in the presence of widespread domestic dog vaccination campaigns
Source: Vet Res. 2022 Dec 12;53:106. doi: 10.1186/s13567-022-01121-1 (PMC9743725; doi:10.1186/s13567-022-01121-1)
Supplement: Supplementary file 5 — Additional file 5. Results of generalized linear models for the incidence of probable jackal rabies cases from models containing the mean domestic dog vaccination coverage from two years before cases occurred. The 95% confidence intervals for the estimates are shown in square brackets []. [file 13567_2022_1121_MOESM5_ESM.docx]

| Model | Dog vaccination coverage – mean of coverage from one and two years before cases occurred (%) – increase of 35% | District area (natural log-transformed) - doubling | Dog population number – increase by 100 dogs | Urban compared to rural | Presence of protected area compared to absence | Human population - increase of 1000 people | Dog population density - increase by 1 dog per km^2^ | Human population density – increase by 10 people per km^2^ | Land classified as savannah (% of district) – increase of 10% |
| --- | --- | --- | --- | --- | --- | --- | --- | --- | --- |
| Jackal  model  1 | 77.3% decrease  [55.2% - 88.8% decrease] |  |  |  |  |  |  |  |  |
| Jackal  model  2 | 75.3% decrease  [53.0% - 87.3% decrease] |  |  | 90.9% decrease  [75.7% - 97.1% decrease] |  |  |  |  |  |
| Jackal  model  3 | 79.3% decrease  [63.2% - 88.7% decrease] | 59.6% increase  [5.4% decrease – 171% increase] | 2.1% decrease  [4.5% decrease – 0.5% increase] | 55.2% decrease  [96.1% decrease – 335% increase] | 93.3% decrease  [76.7% - 98.2% decrease] | 0.1% decrease  [0.9% decrease – 0.7% increase] |  |  | 36.3% increase  [0.5% decrease – 87.7% increase] |
| Jackal  model  4 | 76.8% decrease  [58.2% - 87.4% decrease] |  |  | 80.2% decrease  [97.7% decrease – 112% increase] | 81.4% decrease  [55.5% - 92.8% decrease] |  | 5.4% decrease  [44.3% decrease – 57.8% increase] | 0.4% decrease  [6.6% decrease – 6.1% increase)] | 25.0% increase  [8.0% decrease – 69.5% increase] |
